# A series of patients with minimal change nephropathy treated with rituximab during adolescence and adulthood

**DOI:** 10.1186/s13104-015-1255-0

**Published:** 2015-06-26

**Authors:** Marinus J Dekkers, Jaap W Groothoff, Robert Zietse, Michiel G H Betjes

**Affiliations:** Department of Nephrology, Erasmus MC, Room H 438, P.O. Box 2040, 3000 CA Rotterdam, The Netherlands; Emma Children’s Erasmus MC, P.O. Box 2040, 3000 CA Rotterdam, The Netherlands; Department of Nephrology, Erasmus MC, P.O. Box 2040, 3000 CA Rotterdam, The Netherlands

**Keywords:** Minimal change nephropathy, Immune suppression dependent, Rituximab

## Abstract

**Background:**

The treatment of immune suppression dependent minimal change nephropathy (MCN) can be challenging and frequently leads to serious complications. In paediatric patients, successful treatment with rituximab is described in steroid-dependent MCN. There is limited information about the potential efficacy of rituximab for the treatment of MCN in adults and adolescence. We describe our experience with rituximab in adolescent and adult patients with immune suppression dependent MCN.

**Results:**

Ten adolescents and adults with immune suppression dependent MCN and therapy related complications were treated with rituximab. At a mean age of 26 years, about 10.5 years after first presentation, they received two doses of rituximab (375 mg/m^2^). Maintenance immunosuppressive medication was stopped. After a mean follow-up of 43 months, three patients had four relapses. Three relapses were successfully retreated with rituximab again, after induction therapy with 60 mg prednisone per day. Rituximab was well tolerated and no infectious complications were recorded.

**Conclusion:**

Treatment with rituximab induces a long-term remission of immune suppression dependent MCN in adolescents and adults. A timely treatment with rituximab could be considered to limit side effects of immunosuppressive medication.

**Electronic supplementary material:**

The online version of this article (doi:10.1186/s13104-015-1255-0) contains supplementary material, which is available to authorized users.

## Background

Minimal change nephropathy (MCN) is the most common cause of childhood onset nephrotic syndrome [1]. MCN usually responds well to prednisone, but up to 70% of the patients have one or more relapses of the nephrotic syndrome [2, 3]. A considerable part may become steroid-dependent and over half of the patients with MCN require additional immunosuppressive therapy [3]. Generally the nephrotic syndrome will go into definite remission after puberty, but up to 25% of the children with MCN will continue to suffer from relapses in adulthood [4]. Patients with frequent relapses of the nephrotic syndrome are exposed to long periods of prednisone and have a high risk of therapy-related complications [4]. Other immunosuppressive medication such as calcineurin inhibitors and cyclophosphamide can reduce the cumulative dose of steroids. However, these second-line medications may have serious side effects or limited effectiveness. In recent years, successful treatment with rituximab was described for MCN in children. After treatment with rituximab there are less relapses of the nephrotic syndrome and other immunosuppressive therapy could be substantially reduced [5–8]. There is limited information about the potential efficacy of rituximab for the treatment of MCN in adults and adolescence [9–16]. We describe a series of patients with immune suppression dependent MCN, treated with rituximab in adolescence and adulthood.

## Results and discussion

The patients (Additional file 1: Table S1) with a mean age of 26 (14–56) years had the first presentation of the nephrotic syndrome in childhood (4 cases), adolescence (4 cases) and adulthood (2 cases). The first presentation of the nephrotic syndrome was at an average age of 16 (3–52) years. The mean duration of the nephrotic syndrome was 10.5 (0.5–23) years.

After treatment of rituximab, there was a mean follow-up of 43 months (16–76 months). In this period, three patients had four relapses. One patient (number 9) with a relapse after 24 months and another patients (number 5) with two relapses (21 and 36 months after initial rituximab treatment) were successfully retreated with rituximab after remission induction therapy with 60 mg prednisone/day. Both patients still have a complete response 37 and 43 months after last retreatment with rituximab respectively.

The fourth patient (number 3) had a relapse after 21 months, but retreatment with steroids and rituximab was unsuccessful. The nephrotic syndrome came into remission with prednisone and cyclosporine maintenance therapy.

In all patients, rituximab treatment lead to a total depletion of circulating B cells for at least 1 month. After 1 month, the B cell counts were not routinely measured.

Rituximab was well tolerated, except for one patient who had an allergic reaction during the first treatment. Although the infusion of rituximab was stopped halfway the peripheral B-cells were fully depleted and the nephrotic syndrome went into complete remission without further need for maintenance immunosuppressive medication. None of the patients had infectious complications after treatment with rituximab. In contrast, all patients had experienced serious side effects of the previous (maintenance) immunosuppressive therapy (Additional file 1: Table S1).

## Discussion

The treatment of immune suppression dependent MCN is challenging. Although the nephrotic syndrome usually responds well to prednisone [17, 18], long-term use has serious complications: obesity, cushingoid habitus, osteonecrosis and cataract. Cyclophosphamide, cyclosporine, mycophenolate mofetil or levamisole are commonly used as second-line treatment [5]. Cyclosporine maintenance therapy is usually effective, but relapses often occur after discontinuation [19]. Cyclosporine can cause renal dysfunction, hypertension and gingival hyperplasia. Mycophenolate mophetil may reduce the frequency of relapses [20], but is its use may be limited by gastrointestinal side effects. Maintenance therapy with levamisole, can reduce the number of relapses, but is not known to induce long term remission. In addition, levamisole is not available in every country and could induce agranulocytosis [5, 21]. Only the use of cyclophosphamide may induce a long term remission of MCN in about half of the patients, but cytotoxicity and the risk of infertility are potential serious side effects [22].

In recent years, a number of publications in paediatric patients have reported that rituximab is an effective treatment in steroid-sensitive, but immune suppression dependent nephrotic syndrome. In these children, rituximab treatment seems to be able to reduce the relapse rate, although the immunosuppressive medication was reduced or even discontinued [5–8, 23–26]. The treatment with rituximab in steroid-resistant nephrotic syndrome is considerable less effective [24, 27].

The data about the efficacy of rituximab for MCN in adult patients is limited, but seem to confirm the positive results in children [9–16]. Our data show that adolescents and adults with immune suppression-dependent MCN have a remarkable good long term remission in response to rituximab treatment. Even more remarkable, long term remission was achieved in patients with immune suppression dependent MCN many years after the first presentation and after different immune suppressive drugs and combinations thereof.

Although the pathophysiology of MCN is unknown, the immune system and especially the T-cells seem to play a role [28]. A circulating factor originating from patient’s T-cells can induce MCN in animals [29]. The regulatory T-cells of patients with MCN are less effective compared with T-cells of healthy. When MCN is in remission, these regulatory T-cells functionally improve [30]. Therefore it is interesting that administration of rituximab, a monoclonal antibody directed against CD20, expressed on B-cells, is an effective therapy. In recent years, it is reported that the B-cell has also regulatory functions. The regulatory B-cell stimulates both effector and regulatory T-cells [31]. In other auto-immune diseases the regulatory T-cell function improves after treatment with rituximab [32]. The mechanism of action is unknown, but maybe B-cell depletion results in a new balance between the various T-cell subsets. This balance may establish even after repopulation of the peripheral B-cell population. A suggested mechanism is that T-cells, that interact with B-cells, are removed simultaneous after treatment with rituximab [33]. Otherwise it is also possible that the B-cell has a direct role in the pathogenesis of MCN, but evidence for this position is lacking.

Beside an immunological explanation, rituximab may have a direct effect in the kidney. A recent study shows that rituximab may stabilize the cytoskeleton of the podocyte [34].

We treated our patients with 2 doses of rituximab with a one- or two-week interval, when nephrotic syndrome was in complete remission. Our experience is in agreement with the notion that rituximab seems to be most effective as a remission maintenance drug after induction therapy to achieve complete remission of the MCN [5]. Published studies vary in their dose and number of doses rituximab to achieve long-term remission of MCN. A single dose of rituximab achieved a good response in a series of 12 patients, described by Kamei et al., but the relapse rate was 75% [7]. Another study observation that the time to relapse of the nephrotic syndrome was longer when initially more than three doses were given [23]. An unanswered question is whether rituximab treatment should be repeated after repopulation of the peripheral B-cell population in order to achieve protracted remission. The nephrotic syndrome relapses usually only after repopulation of the peripheral B-cell population [5, 6]. However a large part of the patients have a sustained complete remission, even after repopulation of peripheral B-cells and we therefore did not follow this strategy [6].

Rituximab was well tolerated in our patients. Only one patient had an allergic reaction and infusion was stopped. In previous studies, about 50% of the patients had some complaints when rituximab was administered, but there was no need to stop administration. These symptoms are usually mild and concerns hypotension, tachycardia, dyspnoea and cutaneous rash [5, 6].

Although rituximab causes an average of 6 months B-cell depletion, infectious complications are scarce [5, 6]. A slight depression of serum immunoglobulins is described [35]. Seldom severe complications are pneumocystis jirovecii pneumonia, severe colitis and pulmonary fibrosis [5, 36, 37]. Progressive multifocal leucoencephalopathy is associated with the use of rituximab, but this complication is never described in children treated with rituximab [35]. Adverse events following treatment of rituximab may be dependent of concomitant use of immunesuppressive agents and underlying conditions [35]. After treatment with rituximab we recorded no infectious complications. These limited side-effects of the rituximab treatment are in contrast to complications the other immunosuppressive medications.

## Conclusion

In conclusion, our series of patients shows a remarkable efficacy of rituximab for the induction of long-term remission of immune suppression dependent MCN in adolescents and adults. In the group of patients with immune suppression dependent MCN, with frequent relapses and therapy related complications, a timely treatment with rituximab could be considered to limit side effects.

## Methods

We describe retrospective our experience with the treatment of rituximab in adolescent and adult patients with biopsy proven MCN, who need maintenance immunosuppressive therapy for sustained remission. All patients had normal renal function. They were treated with rituximab because of serious complications due to immunosuppressive medication, frequent relapses or at patients request to stop maintenance immunosuppressive medication. When the nephrotic syndrome was in remission with immunosuppressive therapy, they were treated with 2 doses of 375 mg/m^2^ rituximab with a 1 (patients 8–10) or 2 (patients 1–7) week interval.

Following treatment with rituximab, the dose of prednisone was tapered rapidly and other immunosuppressive medication was stopped within 1 month after the last administration of rituximab.

### Consent

Written informed consent for publication of this case series was obtained from all ten patients. A copy of the written consent is available for review by the Editor-in-Chief of this journal.

## Additional file

**Additional file 1: Table S1.** Patients characteristics, treatment, and complications.

## Abbreviation

MCN: minimal change nephropathy
