# Supplementary material for: A series of patients with minimal change nephropathy treated with rituximab during adolescence and adulthood
Source: BMC Res Notes. 2015 Jun 26;8:266. doi: 10.1186/s13104-015-1255-0 (PMC4482035; doi:10.1186/s13104-015-1255-0)
Supplement: Supplementary file 1 — Additional file 1: Table S1. Patients characteristics, treatment, and complications. [file 13104_2015_1255_MOESM1_ESM.doc]

Table S1. Patients characteristics, treatment, and complications

| patient | sex | Age first NS | Previous treatment | Relapses before RTx treatment | Treatment at the time of RTx | Age RTx treatment | Time from diagnosisto RTx treatment  (years) | Duration follow-up after RTx  (months) | Relapses of NS after RTx  (months) | Complications of prednisone and other immunosuppressive medication |
| --- | --- | --- | --- | --- | --- | --- | --- | --- | --- | --- |
| 1 | M | 11 | prednisone  cyclophosphamide  cyclosporine | 8 | mycophenolate mofetil | 22 | 11 | 30 | none | obesity, cushingoid habitus, striae |
| 2 | M | 17 |  | 3 | prednisone  cyclosporine | 21 | 4 | 21 | none | striae, osteonecrosis shoulder |
| 3 | M | 3 | levamisole | >20 | prednisone  cyclosporine | 22 | 18 | 21 | 21 | worsening of psychosis, obesity,  cushingoid habitus, striae, osteoporosis |
| 4 | M | 17 | prednisone | 2 | cyclosporine | 24 | 7 | 40 | none | cushingoid habitus, acne |
| 5 | M | 3 | prednisone  cyclophosphamide  cyclosporine | 18 | tacrolimus mycophenolate mofetil | 26 | 23 | 73 | 21 and 36 | cushingoid habitus, growth retardation  cataract, osteonecrosis knee, gingival overgrowth |
| 6 | M | 29 | mycophenolate mofetil  cyclophosphamide | >10 | prednisone  cyclosporine | 47 | 18 | 19 | none | dyslipidemia, cataract  systemic Bartonella Henselae infection  renal dysfunction, obesity  obstructive sleep apneua syndrome |
| 7 | F | 52 | prednisone  cyclosporine | 5 | cyclosporine | 56 | 3 | 16 | none | steroid induced diabetes mellitus, abscess leg  toxic megacolon due to clostridium difficile infection, Pneumocytis jirovecii pneumonia |
| 8 | M | 3 | prednisone  cyclophosphamide  cyclosporine | 26 | tacrolimus  mycophenolate mofetil | 17 | 14 | 76 | none | striae, cushingoid habitus , morbid obestity  depression |
| 9 | M | 15 | prednisone  cyclosporine | 3 | cyclosporine | 15 | 0,5 | 67 | 24 | morbid obesity, striae, acne |
| 10 | F | 7 | prednisone  cyclosporine  levamisole | 7 | mycophenolate mofetil | 14 | 7 | 66 | none | gingival hypertrophy, morbid obesity |

NS=nephrotic syndrome, RTx=rituximab
